# mKikGR, a Monomeric Photoswitchable Fluorescent Protein

**DOI:** 10.1371/journal.pone.0003944

**Published:** 2008-12-15

**Authors:** Satoshi Habuchi, Hidekazu Tsutsui, Anna B. Kochaniak, Atsushi Miyawaki, Antoine M. van Oijen

**Affiliations:** 1 Department of Biological Chemistry and Molecular Pharmacology, Harvard Medical School, Boston, Massachusetts, United States of America; 2 Laboratory for Cell Function Dynamics, Brain Science Institute, RIKEN, Wako, Saitama, Japan; 3 Graduate School of Science and Engineering, Tokyo Institute of Technology, Tokyo, Japan; 4 Graduate Program in Biophysics, Harvard University, Cambridge, Massachusetts, United States of America; Massachusetts Institute of Technology, United States of America

## Abstract

The recent demonstration and utilization of fluorescent proteins whose fluorescence can be switched on and off has greatly expanded the toolkit of molecular and cell biology. These photoswitchable proteins have facilitated the characterization of specifically tagged molecular species in the cell and have enabled fluorescence imaging of intracellular structures with a resolution far below the classical diffraction limit of light. Applications are limited, however, by the fast photobleaching, slow photoswitching, and oligomerization typical for photoswitchable proteins currently available. Here, we report the molecular cloning and spectroscopic characterization of mKikGR, a monomeric version of the previously reported KikGR that displays high photostability and switching rates. Furthermore, we present single-molecule imaging experiments that demonstrate that individual mKikGR proteins can be localized with a precision of better than 10 nanometers, suggesting their suitability for super-resolution imaging.

## Introduction

Green fluorescent proteins (GFPs) and GFP-like fluorescent proteins have found extensive use in molecular and cellular biology [1]–[4]. Recently, photoswitchable fluorescent proteins have been reported [5]–[13] whose fluorescence properties can be altered upon illumination at specific wavelengths. The controlled photoconversion of these proteins provides unique opportunities to mark and track selected molecules in cells and organelles [14], [15]. To gain access to molecular dynamics on short timescales, two important requirements are an efficient and rapid photoswitching into the ‘on’ state, and a bright fluorescence after switching.

Another promising application of photoswitchable proteins is their use in super-resolution microscopy. This technique relies on the stochastic photoactivation and localization of single molecules (PALM, STORM, FPALM), in which a fluorescence image is constructed from the high-accuracy localization of individual fluorescent molecules that are switched on and off optically [16]–[20]. Since the localization accuracy is determined by the total photon number detected from individual molecules, the brightness and photostability of the photoswitchable molecules represent the key parameters to achieve high spatial resolution [21].

KikGR, a mutant of a fluorescent protein cloned from the stony coral *Favia favus*, emits bright green fluorescence in its initial state, but is switched into a red emitting species upon illumination with UV or violet light [11]. Since the photoswitching of KikGR is efficient (*φ*_sw_ = 4.7×10^−3^) and the red emitting KikGR shows a bright fluorescence (*φ*_fl_^R^ = 0.65), this protein could be an excellent fluorescent tag for both selective cell labeling and photoactivation localization microscopy. However, KikGR needs to form a homotetrameric complex to become fluorescent similar to other GFP-like proteins [5], [22]. The necessity for a noncovalent oligomerization limits its use in applications with protein fusions.

Here we report a monomeric version of KikGR, mKikGR. We characterize the spectroscopic properties of mKikGR, and show that the protein retains the advantageous spectroscopic properties of KikGR including efficient switching and bright fluorescence.

## Results and Discussion

### Construction of mKikGR

**Figure 1:**
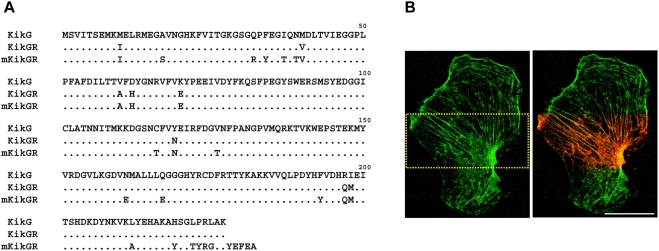
Construction of mKikGR. (A) Amino-acid sequences for KikG, KikGR, and mKikGR. (B) Rat primary astrocyte expressing mKikGR-βactin. Images show before (left) and after (right) local photoswitching by irradiating 405 nm laser over the yellow-boxed region. Cells are cultured at 37°C.

We performed a site-directed random mutagenesis approach on KikGR. The products were also subjected to error-prone PCR to introduce additional variation at other positions that might affect the photoconversion reaction or protein folding. *E. coli* cells transformed with plasmids carrying the mutagenized DNA were plated and screened for photoswitching behavior using a home-made image analyzing system. Then, protein samples were subjected to pseudo-native PAGE analysis to examine their oligomeric state. The directed evolution of KikGR towards a monomeric version was achieved after 15 cycles of mutagenesis. The introduction of 21 mutations, some of which are located in the tetramer interfaces, transformed KikGR into a monomeric version, mKikGR (Figure 1A). We determined the absolute molecular mass of mKikGR to be 30.0 kDa by analytical equilibrium ultracentrifugation analysis. The similarity of this value to that deduced from the primary structure of the protein, 26.5 kDa, confirms its monomeric nature. A mammalian expression plasmid was generated that encodes a chimeric protein comprising mKikGR and human β-actin. Twelve hours post-transfection into primary cultured astrocytes , stress fibers were clearly visible and locally highlighted (Figure 1B).

### Spectroscopic characterization

**Figure 2:**
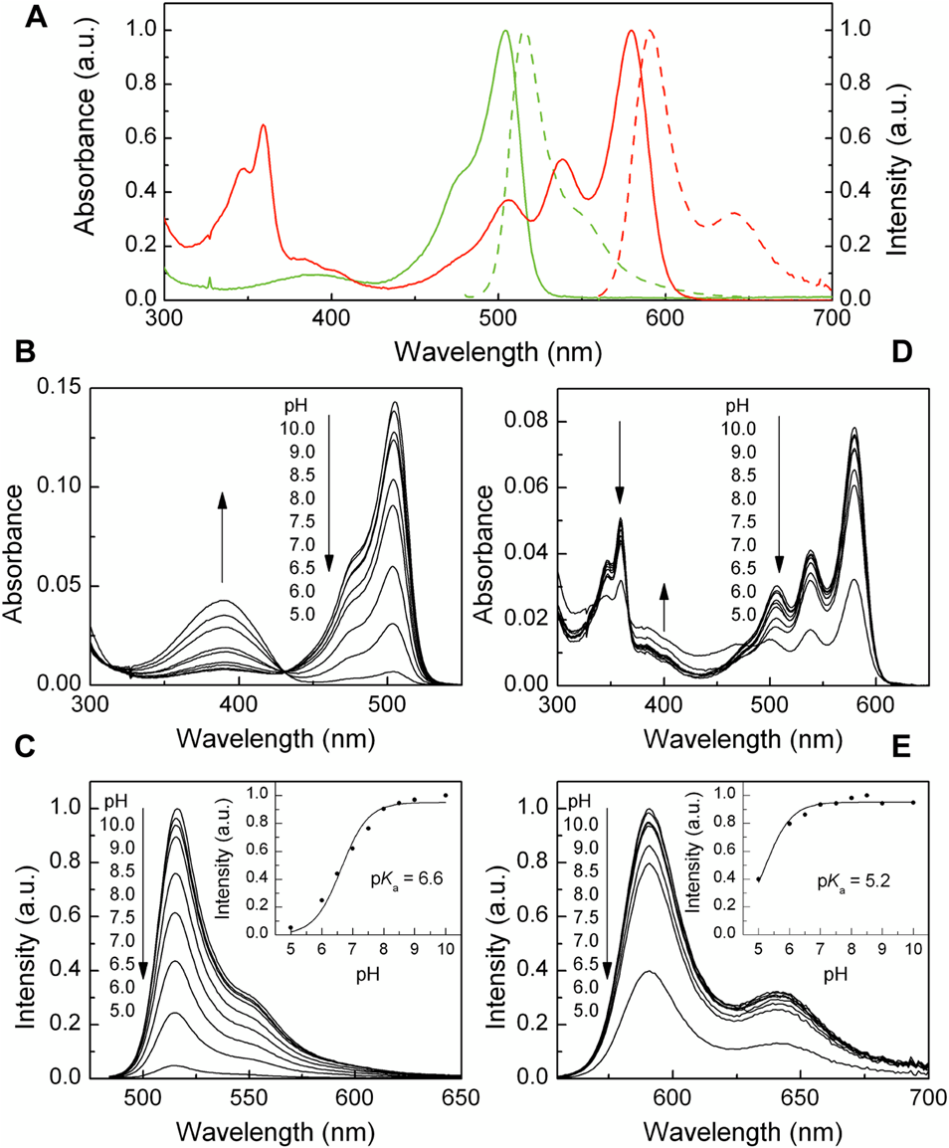
Spectroscopic properties of the green and red form of mKikGR. (A) Normalized absorption (solid line) and fluorescence (dashed line) spectra of the green mKikGR (4.7 µM, green line) and red mKikGR (4.7 µM, red line). The red mKikGR was obtained by illuminating at 405 nm (12 mW cm^−2^) for 90 minutes. Fluorescence spectra of the green and red mKikGR were measured with 475 nm and 555 nm excitation, respectively. All measurements were performed at pH 8.0. (B, D) pH dependence of absorption spectra of the green (B) and red (D) mKikGR (4.7 µM). (C, E) pH dependence of fluorescence spectra of the green (C) and red (E) mKikGR (4.7 µM). Fluorescence spectra of the green and red mKikGR were measured with 475 nm and 555 nm excitation, respectively. (Inset) Peak fluorescence intensities at different pH. The solid lines show fitting with the Henderson-Hasselbalch equation.

The absorption spectrum of mKikGR displays two distinct maxima, at 505 nm (*ε* = 4.9×10^4^ M^−1^ cm^−1^) and 390 nm (*ε* = 1.2×10^4^ M^−1^ cm^−1^) (Figure 2A, green solid line). The pH dependence of these two absorptions (Figure 2B) demonstrates that the 505-nm band corresponds to the deprotonated form of the mKikGR chromophore, and the band at 390 nm corresponds to the protonated form. While the protonated form of the chromophore was nearly non-fluorescent (data not shown), the deprotonated form of the chromophore showed bright fluorescence when excited at the absorption band of the deprotonated form (*φ*_fl_^G^ = 0.69; see Figure 2C). The p*K*_a_ of the chromophore was determined from the pH dependence of the fluorescence intensity (p*K*_a_ = 6.6, see Figure 2C inset).

Upon illumination at 405 nm, the initial state of the chromophore (green mKikGR) was converted into a red mKikGR, in which both the absorption and fluorescence spectra were red shifted. The absorption spectrum of the red mKikGR displays peaks at 580 nm (*ε* = 2.8×10^4^ M^−1^ cm^−1^) and 359 nm (*ε* = 1.8×10^4^ M^−1^ cm^−1^) (Figure 2A, red solid line). Bands at the same wavelengths are present in a fluorescence excitation spectrum detected at 600 nm (data not shown). This result together with pH dependence of the absorption spectrum of the red mKikGR (see Figure 2D) suggests that both bands are attributed to the deprotonated form of the chromophore. The 359-nm band could be attributed to a higher excited state of the red mKikGR chromophore. The red mKikGR displayed brightness equal to its green counterpart. The fluorescence spectrum of the red mKikGR showed a peak at 591 nm (*φ*_fl_^R^ = 0.63). The p*K*_a_ of the red mKikGR is lower than that of the green mKikGR (p*K*_a_ = 5.2, see Figure 2E). The spectroscopic properties of mKikGR (see Table 1) are similar to those of tetrameric KikGR.

**Table 1:** Spectroscopic properties of monomeric photoswitchable proteins which change color on illumination.

	φ_sw_	φ_fl_^in^	ε^in^	φ_fl_^sw^	ε^sw^	φ_bl_
PS-CFP	N.A.	0.16	3.4×10^4^	0.19	2.7×10^4^	N.A.
mEosFP	N.A.	0.64	6.7×10^4^	0.62	3.7×10^4^	3.0×10^−5^
Dendra	N.A.	0.72	2.1×10^4^	0.70	2.0×10^4^	N.A.
mKikGR	7.5×10^−3^	0.69	4.9×10^4^	0.63	2.8×10^4^	6.5×10^−6^
KikGR^*^	4.7×10^−3^	0.70	2.8×10^4^	0.65	3.3×10^4^	N.A.

φ_sw_: quantum yield of photoswitching, φ_fl_^in^: fluorescence quantum yield of an initial state, ε^in^: molar extinction coefficient of an initial state, φ_fl_^sw^: fluorescence quantum yield of a photoswitched state, ε^sw^: molar extinction coefficient of a photoswitched state, φ_bl_: photobleaching quantum yield of the photoswitched state. ^*^The spectroscopic properties of KikGR are also listed as a reference.

### Kinetics of photoswitching

**Figure 3:**
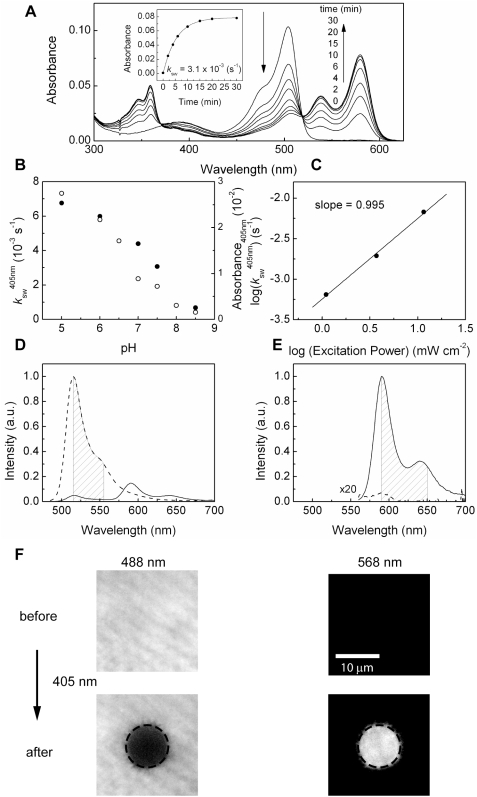
Kinetics of photoswitching from the green to red mKikGR. (A) Time course of the absorption spectra of mKikGR (4.7 µM, pH 7.5) on illumination at 405 nm (12 mW cm^−2^). (Inset) Time course of the peak absorbance of the red mKikGR (580 nm). The solid line shows the fitting with a first-order kinetic model. (B) pH dependence of the rate of the photoswitching (solid circles, left axis) and absorbance at 405 nm (open circles, right axis). (C) Excitation power dependence of the photoswitching. The rates were determined at pH 5.0. (D, E) Fluorescence spectra of mKikGR before (dashed lines) and after (solid lines) photoswitching. The spectra were measured with 475 nm (D) excitation and 555 nm (E) excitation. (F) The top panels show fluorescence images of the green (left) and red (right) mKikGR embedded in the thin film of polyacrylamide gel (10 µM, pH 7.0). The wavelength regions that emission filters transmit are indicated by shadow in Fig. 2D (green) and 2E (red). The region indicated by circle was illuminated with 405 nm light (1 sec, 6.3 W cm^−2^). The bottom panels show fluorescence images of the green (left) and red (right) mKikGR.

Figure 3A shows the time evolution of the absorption spectrum of mKikGR at pH 7.5 upon illumination at 405 nm (12 mW cm^−2^). The absorption band of the green mKikGR was reduced and concomitantly the absorption band of the red mKikGR increased. The spectra display isosbestic points at 519 and 371 nm, indicating that the switching of the chromophore can be described as a simple conversion between two states. Time evolutions of the peak absorption of the red mKikGR can be fitted by a first-order kinetic model (Figure 3A inset), giving a rate of photoswitching of 3.1×10^−3^ s^−1^. SDS/PAGE experiments demonstrated fragmentation of mKikGR upon illumination. While the green mKikGR showed a single band at 28 kDa, the red mKikGR showed two bands at 18 and 10 kDa (data not shown). This result strongly suggests that the β-elimination reaction observed in the photoactivatable protein Kaede also occurs in the photoswitching of mKikGR [5], [23], [24].

**Figure 4:**
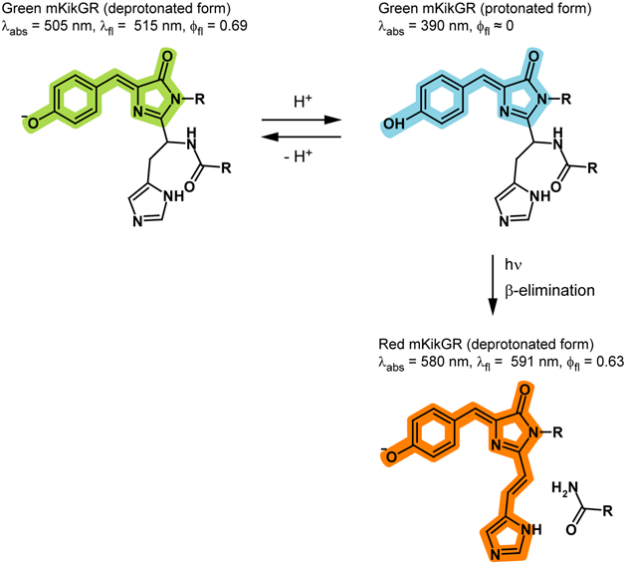
Reaction scheme of the photoswitching from the green to red mKikGR.

The rate of photoswitching depends significantly on pH. The switching rate at pH 5.0 (*k*_sw_^405 nm^ = 6.8×10^−3^ s^−1^) is about ten-fold faster than that at pH 8.0 (*k*_sw_^405 nm^ = 6.7×10^−4^ s^−1^) (Fig. 3B). Furthermore, the photoswitching rate is nearly proportional to the absorption of the protonated form at 405 nm (Figure 3B). Those results suggest that the photoswitching reaction initiates from the protonated state of the green mKikGR [5], [10]. The quantum yield of the photoswitching (*φ*_sw_) is 7.5×10^−3^, as calculated from the absorption cross-section of mKikGR at 405 nm (3.83×10^−17^ cm^2^), the rate of the photoswitching, and the illumination intensity of the 405 nm light (11.6 mW cm^−2^). This value is somewhat larger than that of kikGR (*φ*_sw_ = 4.7×10^−3^) [11]. The rate of the photoswitching at a constant pH increases linearly with the illumination power of the 405 nm light (Figure 3C), suggesting that the photoswitching reaction occurs through a one-photon excitation mechanism. The reaction scheme of the photoswitching is drawn in Figure 4 [24], [25].

Figure 3D and 3E show fluorescence spectra of mKikGR excited at 475 nm (Figure 3D) and 553 nm (Figure 3E) before (broken lines) and after photoswitching (solid lines) by illuminating at 405 nm. The photoswitching resulted in a 560-fold increase in the ratio of red to green fluorescence (R/G), from 0.043 to 24.0. Figure 3F shows fluorescence images of mKikGR (10 µM) embedded in a polyacrylamide gel. After a single pulse (1 sec) illumination with 405 nm light (6.3 W cm^−2^), a significant decrease of the green fluorescence (bottom left) and increase of the red fluorescence (bottom right) were observed. The R/G ratio changed from 0.00248 to 0.90363, a 364-fold increase after the single pulse illumination. The fast switching is as efficient as that observed in the tetrameric Kaede protein [5].

### Single-molecule photoswitching

**Figure 5:**
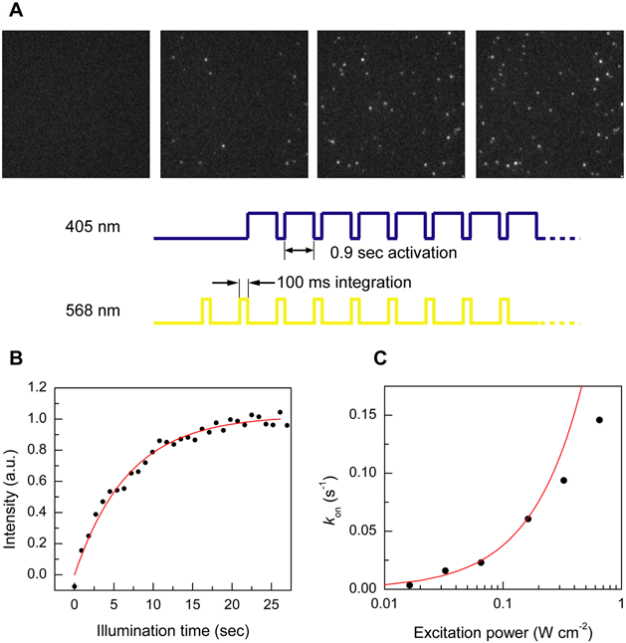
Photoswitching of mKikGR at the single-molecule level. (A) Fluorescence images of individual red mKikGR molecules embedded in polyacrylamide gel (100 pM, pH 7.0). The images were recorded with 568 nm excitation (130 W cm^−2^, 100 ms integration, 1 Hz). The sample was illuminated with 405 nm light (33 mW cm^−2^) between 568 nm pulses. The images were recorded after 0, 1.8, 8.1, and 22 seconds total illumination time with 405 nm light. (B) Time course of integrated intensities of the images. The solid line shows fitting with a first order kinetic model. (C) Excitation power dependence of the photoswitching rates determined from the single-molecule measurements. The solid line shows theoretical switching rate calculated from bulk experiments.

Figure 5A shows fluorescence images of individual red mKikGR molecules (100 pM, 568 nm excitation) embedded in a polyacrylamide gel. The images were recorded every second with 100 ms integration at 568 nm excitation, and the same area was illuminated with the switching light (405 nm) between each frame (see Figure 5A bottom). Since only the red mKikGR is excited at 568 nm, no fluorescent spots were observed before illumination at 405 nm (Figure 5A, first image). Bright fluorescent spots appeared immediately after the first pulse of the 405 nm light (Figure 5A, second image). The number of the spots increased with the 405 nm pulses (Figure 5A, third and fourth image). Integrated intensities of the field of view can be fitted with a first-order kinetic model (Figure 5B). The rates of the photoswitching obtained from the single-molecule experiments (Figure 5C, circles) agree well with those expected from the bulk-measured switching rate constant (Figure 5C, solid line; switching rate constant as obtained from Figure 3A, inset). At higher excitation power, the photoswitching rates slightly deviate from the calculated values. Since the red mKikGR has an absorption at 405 nm (see Figure 2A), the apparent decrease in the switching rates at higher illumination power could be due to photobleaching of the red mKikGR. An alternative interpretation is that the switching reaction occurs through an intermediate state, which has relatively long lifetime [26], [27].

### Photobleaching of red mKikGR

**Figure 6:**
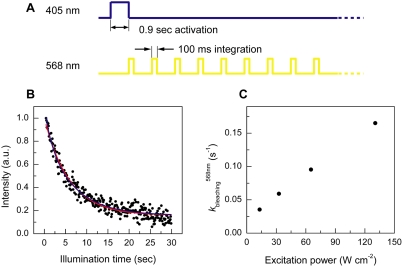
Single-molecule photobleaching of mKikGR. (A) Schematic illustration of experimental configuration. The excitation power of the 405 nm and 568 nm lights were set at 6.3 and 130 W cm^−2^, respectively. (B) Time course of the fluorescence intensity of the red mKikGR embedded in polyacrylamide gel (100 pM, pH 7.0). The red and blue lines show a single- and double-exponential fit. (C) Excitation power dependence of the photobleaching rates of the red mKikGR.

The green mKikGR molecules were photoswitched into the red mKikGR with a single pulse of 405 nm light, and fluorescence images of individual red mKikGR molecules were recorded every second with 568 nm excitation (Figure 6A). Figure 6B shows a time course of integrated fluorescence intensities. The intensity trace was fitted with either single- or double-exponential functions (Figure 6B red and blue lines). A photobleaching rate was calculated from an average time constant of the fitting curve (Figure 6B). Although photobleaching of the red mKikGR showed complicated kinetics, the bleaching rates increased almost linearly with increasing the excitation power of the 568 nm light. (Figure 6C). Photobleaching quantum yield of the red mKikGR (*φ*_bleaching_) was determined to be *φ*_bleaching_ = 6.47×10^−6^, which was calculated from the rate of photobleaching (*k*_bleaching_ = 0.165 s^−1^), the abosorption cross-section (*σ*_568 nm_ = 6.84×10^−17^ cm^2^), and the excitation power (*I* = 130 W cm^−2^). This value is more than four times smaller than that of mEosFP (≈3.0×10^−5^) [10], a monomeric photoswitchable fluorescent protein which changes the color of the fluorescence from green to red upon illumination with UV light. The number of photons that we are able to detect from a single red mKikGR molecule is about 9.7×10^3^, which is calculated from the photobleaching quantum yield (*φ*_bleaching_ = 6.47×10^−6^), and the quantum yield of fluorescence (*φ*_fl_^R^ = 0.63), assuming that the photon detection efficiency (*η*_det_) of the setup is 0.1.

### Superresolution position determination of individual mKikGR proteins

The monomeric mKikGR displays bright fluorescence (*φ*_fl_^R^ = 0.63), a large quantum yield of photoswitching (*φ*_sw_ = 7.5×10^−3^), and a low quantum yield of photobleaching (*φ*_bleaching_ = 6.47×10^−6^). Those spectroscopic properties of mKikGR make the protein a candidate for photoactivation localization microscopy (PALM) [16]. The principle of PALM relies on the stochastic activation of spatially well-separated fluorescent proteins and the ability to determine their location with a precision much better than the diffraction limit. When after localization the proteins are photobleached and new ones nearby are activated, their relative positions are obtained and a local structure can be reconstructed [16]–[20]. The resolution of the resultant reconstructed image is ultimately determined by the accuracy of position determination of the individual molecules, a value that is limited by the number of photons that can be emitted by a single molecule before photobleaching.

**Figure 7:**
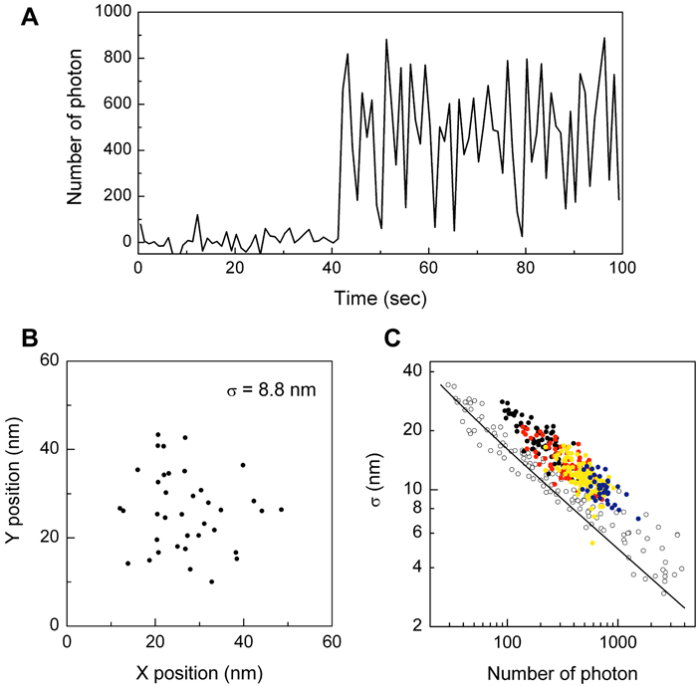
Localization accuracy obtained from mKikGR molecules. (A) Fluorescence intensity trajectory of a single mKikGR molecule. The trajectory was constructed from single-molecule images of the red mKikGR (100 ms integration, 1 Hz). (B) Centroid positions obtained from single-molecule images of the red mKikGR. (C) Relationship between the localization accuracy and the total number of photons obtained from individual red mKikGR molecules (circle). The images were recorded with 568 nm excitation at the power of 13 (black circle), 33 (red circle), 65 (yellow circle), and 130 (blue circle) W cm^−2^. The open circle shows the relationship between the localization accuracy and the total number of photons obtained from individual qdots. The solid line shows theoretically calculated localization accuracy using equation 1.

The significantly improved photobleaching stability of mKikGR compared to other monomeric photoactivatable proteins suggests that use of mKikGR in localization microscopy could result in a further improvement of resolution. Here we determined localization accuracies obtained from individual mKikGR molecules. Figure 7A shows a typical fluorescence intensity trajectory of a single mKikGR in the red state, recorded every second with 100 ms integration time (excited at 568 nm). The molecule was photoswitched from the green to red at *t* = 41 sec. The trajectory showed large intensity fluctuations, which corresponds to blinking and is typical for fluorescent proteins [28]. We fitted the single-molecule fluorescence spot with a 2D Gaussian profile, determined its centroid, and repeated this every second until photobleaching occurred. Figure 7B shows the distribution of the centroid positions determined from the time series of images of the single mKikGR protein whose intensity trace is shown in Figure 7A. The standard deviation of the set of positions is 8.8 nm and represents the localization accuracy obtained from a single 100-ms acquisition.

Figure 7C (solid circles) shows the relationship between the localization precision calculated from the sets of centroid positions derived from a single mKikGR and the total number of photons present in a single-molecule image. We also analyzed individual qdot molecules (qdot655) as a reference (Figure 7C, open circles). The solid line in Figure 7C is a theoretical localization precision calculated by the equation [21],
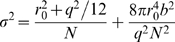
(1)where *r*_0_ is the standard deviation of the point spread function (150 nm), *N* is the total number of photons collected, *q* is the size of an image pixel (166 nm), and *b* is the background noise per pixel (0.5 photon per pixel). The theoretically calculated localization precision agrees well with the experimental data. The localization precision obtained from mKikGR molecules are about 40% less than that of the reference sample. This is partially due to the less bright fluorescence of mKikGR as compared with the reference sample which results in a higher background signal. The blinking behavior of mKikGR (see Figure 7A) also affects the signal to noise ratio which limits the localization precision.

Our results demonstrate that mKikGR is suitable for localization microscopy using fluorescent proteins. The small photobleaching yield (6.5×10^−6^) is an advantage of mKikGR over reversible photoswitchable fluorescence proteins such as Dronpa which shows a photoswitching yield (from the bright to the dark state) of 3.2×10^−4^. Although it was demonstrated that the use of Dronpa is advantageous in photoactivation localization microscopy [20], [29] due to its efficient switching from the bright to the dark state [8], [30], [31], this rapid switching results in lower number of photons collected in single images that result in a limited spatial resolution. The large number of photons obtained from red mKikGR is useful not only in photoactivation localization microscopy, but also for single-molecule tracking. A localization precision of about 30 nm can be achieved with 100 collected photons (see Figure 7C). This result together with the total number of photons that can be collected from a single red mKikGR suggest that one should be able to record 100 images with 30 nm localization precision, which meets the requirements in most of single-molecule tracking experiments.

We have produced a highly photostable, monomeric photoactivatable fluorescent protein, and characterized its photophysical properties. Its monomeric form allows it to be used as a highly specific and stable fluorescent tag. We have demonstrated that its increased photostability can be used to improve resolution of position determination, illustrating its applicability to superresolution localization microscopy. An important future direction will be the expansion of the arsenal of photoswitchable proteins to different emission wavelengths allowing for multicolor imaging.[5]

## Materials and Methods

### Mutagenesis

Site-directed semi-random mutations were introduced as described previously [32]. Multiple degenerative primers, greater than ten in some cases, were used together for reactions. Additional random mutations were introduced using the error-prone PCR [33].

### Analytical ultracentrifugation

Sedimentation equilibrium experiments were performed using a Beckman XL-1 analytical ultracentrifuge at 20°C. Absorbance was measured at the maximum wavelength as a function of radius at 25,000 rpm.

### Protein expression, purification, pH titration, SDS/PAGE

Recombinant mKikGR protein was expressed in *E. coli*. The protein was purified with Ni-NTA chromatography (Qiagen) [5], [34], and dialyzed into 50 mM Tris at pH 8.0, 300 mM NaCl, 1 mM DTT, and 10% Glycerol. The solutions for pH titration contained 125 mM KCl, 20 mM NaCl, 0.5 mM CaCl_2_, 0.5 mM MgCl_2_, and 25 mM of one of the buffers – acetate, Mes, Hepes, Tris, or bicarbonate. 1 N NaOH and 1 N HCl solutions were used to obtain the correct pH. SDS/PAGE analysis was done with 12% polyacrylamide gels (Bio-Rad).

### Ensemble spectroscopy

Absorption spectra were measured with a spectrophotometer (PerkinElmer) using a UV-transparent cuvette (Brandtech). For calculation of molar extinction coefficients, protein concentrations were determined using a Bradford assay kit (Bio-Rad) with BSA as a standard. Fluorescence spectra were recorded with a fluorimeter (Photon Technology International) using a quartz cuvette. The fluorescence quantum yields of the green and red mKikGR were determined using fluorescein (*φ*_fl_ = 0.95 in 0.1 M NaOH) and sulforhodamine 101 (*φ*_fl_ = 0.98 in ethanol) as references. We photoswitched mKikGR (500 µl, 5×10^−6^ M) by illuminating the samples with a diode-pumped solid-state laser at 405 nm (CrystaLaser) at a power of 1.2–12 mW cm^−2^.

### Single-molecule imaging

10 µl of a solution containing 100 pM mKikGR and 15% polyacrylamide was deposited on a clean coverslip. A second coverslip was placed on top of the sample to produce a thin film of mKikGR-containing gel. The samples were mounted on an inverted microscope (IX-71, Olympus). 568-nm light from an Ar-Kr ion laser (Innova 70C-Spectrum, Coherent Inc.) and 405-nm light was focused into the back-focal plane of the microscope objective (PlanApo, N.A. = 1.45, Olympus) to create a collimated beam, resulting in the illumination of a circular area with a diameter of 50 µm at the sample plane. The fluorescence of the samples was collected by the same objective and, after passing a dichroic mirror (T585lp) and emission filter (ET620/60), focused onto an EM-CCD camera (iXon, Andor Technology). The images were further magnified 1.6 times with a lens before the camera, resulting in a pixel size of 166 nm×166 nm. While the fluorescence images of the red mKikGR molecules were recorded with the 568 nm excitation light (100 msec integration, 1 Hz), the 405 nm light was introduced into the microscope between the 568 nm pulses to photoswitch mKikGR molecules from the green to red form. The illumination timing was controlled by shutters (Uniblitz).

A quantum dot sample was prepared by depositing 1 pM Qdot655 (Molecular Probes) in TE buffer (20 mM Tris at pH 7.5, 2 mM EDTA) on a clean coverslip. The images were recorded in a similar way as for the mKikGR sample. The 488-nm line of the Ar-Kr laser was used as an excitation source.

The fluorescence images were analyzed using MATLAB script designed and written in house for particle detection and localization.
